# Managing Early Childhood Caries in Asia-Pacific Region

**DOI:** 10.1016/j.identj.2025.100945

**Published:** 2025-09-22

**Authors:** Faith Miaomiao Zheng, Chun-Pin Lin, Olabode Ijarogbe, Elham Kateeb, Hiroshi Ogawa, Syed Mahmood Shah, Fernando Fernanodez, Ting-Chen Chen, Ting-Yi Renn, Chun Hung Chu, Wei-Jen Chang

**Affiliations:** a Faculty of Dentistry, The University of Hong Kong, Hong Kong, China; b Department of Dentistry, National Taiwan University Hospital, Taipei, Taiwan; c Graduate Institute of Clinical Dentistry, School of Dentistry, National Taiwan University, Taipei, Taiwan; d Asia Pacific Dental Federation, Manilla, Philippine; e Department of Restorative Dentistry, Faculty of Dental Sciences, College of Medicine, University of Lagos, Lagos, Nigeria; f Oral Health Research and Promotion Unit, Al-Quds University, Jerusalem, State of Palestine; g Division of Preventive Dentistry, Department of Oral Health Science, Graduate School of Medical and Dental Sciences, Niigata University, Niigata, Japan; h Department of Orthodontics, Muhammad Dental College, Mirpurkhas, Sindh, Pakistan; i Department of Oral Health, Ministry of Health and Welfare, Taiwan; j School of Dentistry, College of Oral Medicine, Taipei Medical University, Taipei, Taiwan; k Dental Department, Shuang-Ho Hospital, Taipei Medical University, Taipei, Taiwan

**Keywords:** Early childhood caries, Asia-Pacific, Oral health promotion, Children

## Abstract

Early childhood caries (ECC) affects about half of preschool children in Asia. However, most preschool children in Asia-Pacific do not receive dental care for their ECC. Untreated ECC causes pain and infection, affecting the growth, development, and quality of life of children. The Asia Pacific Dental Federation (APDF) emphasizes the need for ECC management to address the prevalence and severity of ECC. The APDF organized an ECC management workshop at the Asia Pacific Dental Congress 2024. An ECC working group was formed by a panel of health experts who reviewed the literature and developed ECC management recommendations for different stakeholders in Asian countries and regions. Drawing on the promise of current ECC management practices from Asia-Pacific, the working group recommends forming partnerships with the government to improve oral health literacy by implementing nationwide annual dental check-ups for pre-schoolers in communities or kindergartens. Policy makers should prioritize oral health in universal health coverage, integrate it into the general healthcare system, and improve access to oral health services. Dental associations should provide guidelines for ECC management, implement school-based oral health programs, and collect data on children's oral health. Oral health professionals should employ minimal invasive interventions, raise awareness, and collaborate with other healthcare providers. Academia should conduct research, translate findings into practice, and offer education and training. Parents should ensure proper oral hygiene, promote a healthy diet, and schedule regular check-ups. Educational institutions should incorporate oral health education and implement preventive measures. These recommendations aim to facilitate the adoption of preventive measures for ECC management.

## Introduction

Oral health is an important part of general health. The World Health Organization (WHO) states that oral health is integral to general health and a key indicator of overall health, well-being and quality of life.^1^ Oral health affects children’s lives and future development. Tooth decay (dental caries) is the most common oral disease. Early childhood caries (ECC) is dental caries in the primary teeth of children under the age of 6.^2^ ECC causes pain and infection, and advanced caries will progress into the tooth pulp and dental abscesses.^3^ If children’s carious teeth remain untreated, the disease leads to tooth loss and compromised dentition. More importantly, compromised dentition significantly affects the child’s nutrition and, consequently, his or her growth, development, and general health.^4^ The local infection developed from ECC may spread and even threaten the child’s life.^5^

**Fig. 1:**
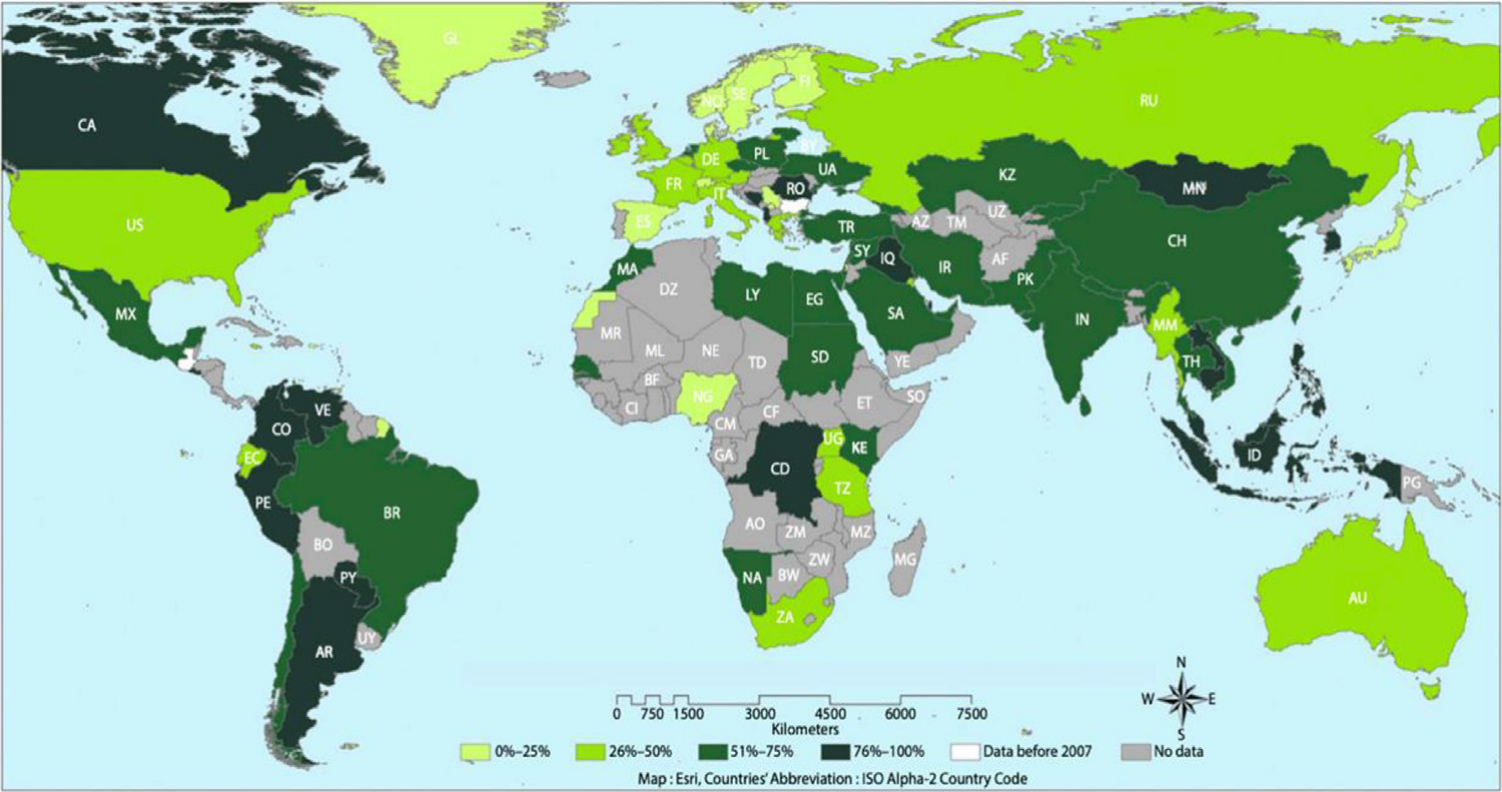
Global prevalence of early childhood caries 2007 to 2017 (adapted from El Tantawi et al., 2018).

The Asia-Pacific region, which makes up more than 50% of the world's population, is home to a significant number of children. In this area, there are approximately 580 million children, out of which 150 million are under the age of 5, emphasizing the vast presence of young lives in this region.^6^ The prevalence of ECC in the Asia-Pacific region is alarmingly high at 68.7% (Figure 1),^7^ indicating a significant dental health concern among young children in these areas.

### Inadequate healthcare coverage

Many countries lack specific oral healthcare coverage for young children, despite their increased dental needs and challenges in accessing dental care. Thailand and Japan stand out as the only 2 Asia-Pacific countries that have integrated oral healthcare into general healthcare services and implemented targeted oral health policies for young children. Their policies both aim to improve children's oral health at an early stage.^8^^,^^9^ Under universal health coverage (UHC), all Thai children aged lower than 12 years old can receive free-of-charge oral health promotion, prevention, and dental treatment at all dental clinics in the public (government) sector.^8^ As a welfare country, Japan established a universal health insurance system earlier in 1961 that covers the entire population. This system allows people to receive dental treatment at relatively low costs including children, with standardized fees applied nationwide.^9^ However, preventive dental services are excluded, as the current health insurance system only covers treatments for existing diseases.^9^

### Access inequities

Limited access to dental care and a shortage of dental professionals in the Asia-Pacific region hinder the timely treatment of ECC, leading to more severe consequences for affected children. However, in countries where there has been improved access to dental care, the prevalence of ECC in children has seen a decrease. Since 2018, the Faculty of Dentistry at the University of Hong Kong has conducted the Jockey Club Children Oral Health Project to serve all Hong Kong kindergarten students. The project includes dental screenings for children, fluoride application when necessary, oral health education for parents, and workshops for teachers.^10^ This initiative aligns with a decrease in caries prevalence among 5-year-olds, from 50.7%^11^ in 2011 to 41.6%^12^ in 2021. As well as in Japan, it is common for kindergartens to have access to dental services.^9^ Dental check-ups are often provided by dentists and dental hygienists who visit the kindergartens. Schoolchildren can receive comprehensive dental treatment at any public or private dental clinic rather than in school.^9^ The prevalence of dental caries among 5-year-olds Japan children decreased from 94.5% in 1957 to 39.0% in 2016.^9^

### Policy-practice gap

**Fig. 2:**
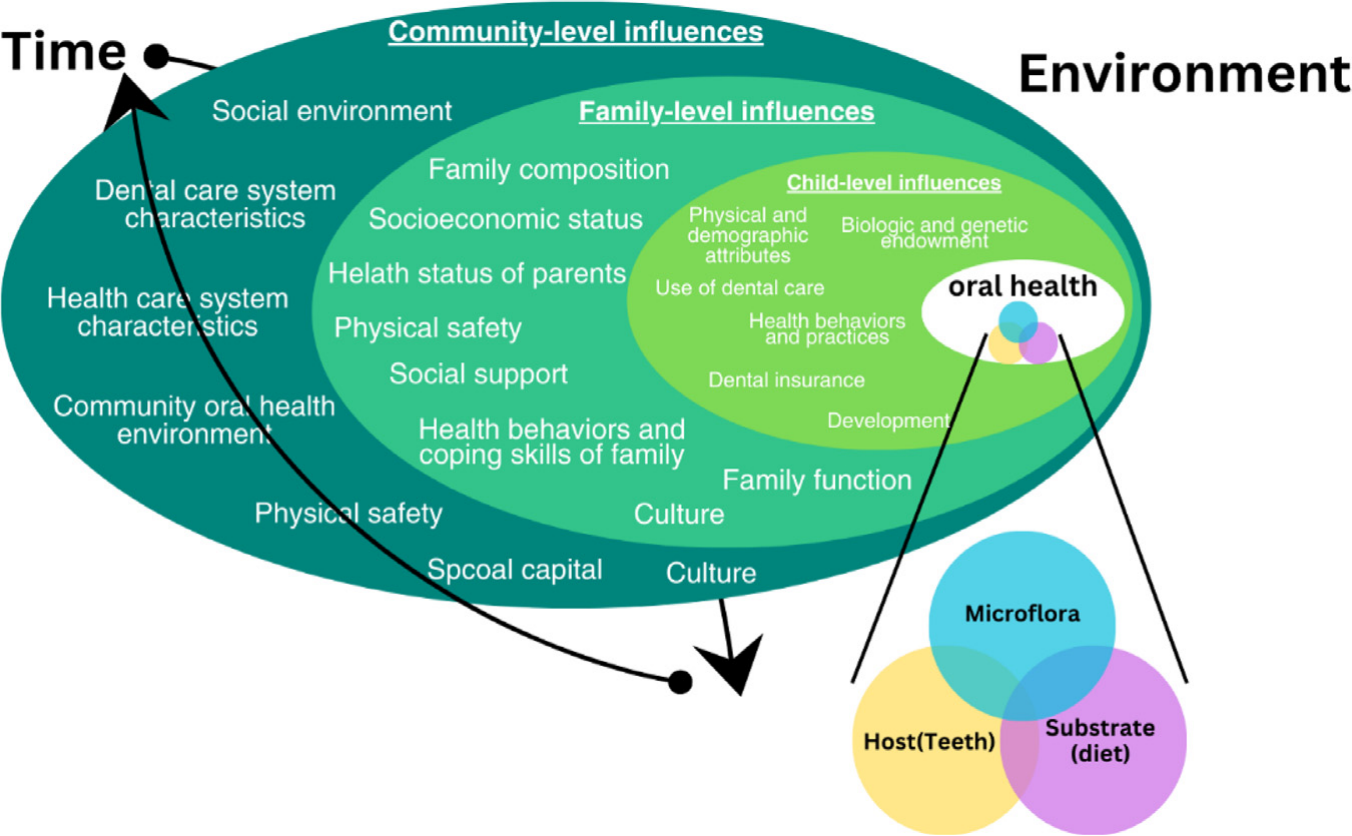
Child, family, and community influences on early childhood caries (adapted from WHO, 2019).

Although WHO guidelines explicitly endorse collaboration among parents at the family level, dental professionals, and policymakers at the community level (Figure 2),^13^ fragmented implementation and limited community engagement continue to result in persistently high rates of ECC across the region. Most Asia-Pacific countries lack effective multi-stakeholder alignment. In contrast, Japan has successfully reduced ECC through integrated policies, including banning added sugar in infant formula, promoting the use of fluoride toothpaste, and actively mobilizing community involvement.

Presently, the Asia-Pacific region faces challenges such as dental personnel shortages, limited resources, budget constraints, disparities, and undervaluation of primary tooth care for children. Collaboration among policy makers, dental associations, professionals, academics, parents or guardians, and educational institutions can enhance dental health among young children. Fostering inter-country/regional cooperation enables knowledge and resource sharing, leading to targeted strategies for ECC reduction and improved children's dental health. These recommendations aim to facilitate ECC management adoption across various stakeholders in the Asia-Pacific region.

## Methods

The Asia Pacific Dental Federation (APDF) recognizes the importance of addressing ECC management due to its high prevalence and severity in the region. To tackle this pressing issue, the APDF organized an ECC management workshop during the Asia Pacific Dental Congress 2024. The workshop aimed to develop recommendations for different stakeholders to promote oral healthcare for young children in the Asia-Pacific region. A working group was hence formed by a panel of health experts in pediatric dentistry and public health. They reviewed the current literature on oral healthcare for young children to identify the challenges in implementing oral healthcare for young children and formulated recommendations for different stakeholders to manage ECC in the Asia-Pacific region. The working group engaged in a series of discussions and consultations with key stakeholders, such as dental professionals, educators, policy makers, and community leaders, to gather insights and feedback on the proposed recommendations. This collaborative approach ensured that the recommendations were both practical and culturally appropriate for the target populations. The working group convened on 2nd May 2024 at the 45th Asia Pacific Dental Congress in Taipei, Taiwan, China to discuss and consent to the recommendations.

### Recommendations for policy makers

This section presents evidence-based recommendations aimed at strengthening ECC management guidelines across the Asia-Pacific region. Given the diversity in resources, health systems, and population needs, local adaptation of these guidelines is crucial. Table summarizes the recommendations for different stakeholders in managing ECC among young children.

**Table:** Recommendations for stakeholders on early childhood caries (ECC) management.

Actions *(stakeholders)*	Rationale
***Policy makers***	
• To include oral health in universal health coverage	*Tackling oral health disparities to ensure accessible and affordable child’s dental care*
• To integrate oral health into the general healthcare system	*Offering a holistic, patient-centered, and multidisciplinary approach to enhance child’s oral and general health*
• To initiate policies to advocate for nutritious dietary choices	*Encouraging healthy eating habits from a young age for lifelong well-being*
• To improve physical access to oral health services	*Minimizing barriers to oral healthcare in remote areas and for those with special needs*
***International and national dental associations***	
• To provide education and interprofessional training	*Optimizing oral care and integrating health services for children*
• To provide guidelines on ECC management	*Advocating patient-cantered prevention-oriented approach in ECC management*
• To advocate early detection of ECC	*Reducing costs and complexity while enhancing clinical outcomes for young children*
• To conduct school-based oral health promotion	*Adopting population-wide upstream approach in tackling ECC*
• To perform annual oral health survey as global surveillance	*Providing evidence to guide and support oral health policy*
***Individual oral healthcare professionals***	
• To adopt early minimal invasive intervention for ECC management	*Reducing costs and complexity while enhancing clinical outcomes for young children*
• To raise awareness on child’s oral health	*Improving child's oral health by equipping parental/caregivers’ oral health knowledge*
•To collaborate with healthcare professionals	*Advocating patient-centred prevention-oriented approach in ECC management*
***Academia***	
• To conduct research improving child’s oral health	*Providing evidence for the best practice on ECC management*
• To translate research findings into practice	*Adopting evidence-based guidelines in dental practices*
• To provide education and training opportunities	*Empowering oral healthcare professionals with up-to-date education and training for ECC management.*
***Parents or guardians***	
• To encourage proper child’s oral hygiene practices	*Ensuring timely and consistent oral healthcare for children*
• To monitor and promote a healthy diet	*Promoting a healthy diet by providing balanced meals*
• To ensure regular dental check-ups	*Monitoring children's oral health status and ensuring timely treatment*
***Educational institutions***	
• To promote oral health education into the curriculum	*Enabling children to acquire early oral health knowledge*
• To integrate preventive measures for ECC management into daily routines	*Ensuring timely and consistent oral healthcare for children*
• To collaborate with dentists and parents for timely children's oral health	*Ensuring timely information exchange regarding child's oral health*

#### To include oral health in universal health coverage (UHC)

UHC seeks to diminish health disparities by guaranteeing that everyone has access to a comprehensive spectrum of health services, including preventive, promotive, curative, rehabilitative, and palliative care. These high-quality interventions aim to meet individuals' needs without imposing financial burdens, regardless of their economic status.^14^ The WHO and the FDI World Dental Federation have proposed to include oral health in UHC to make oral healthcare accessible, available and affordable to all including young children.^15^^,^^16^

Including young children in UHC is crucial for several key reasons. Early intervention leads to time and cost savings, as it prevents severe health problems and reduces the need for expensive treatments later in life. Additionally, this coverage promotes healthy development and contributes to general health, ensuring that children have the best possible start and reach their full potential in life.

UHC including children’s oral health needs strong government support, better health financing schemes, and efficient management of the oral healthcare workforce. Focusing on health management and community-based caregivers is essential.^17^ By providing better oral healthcare facilities, we can improve educational and employment outcomes and help prevent people from falling into poverty.^17^ For example, in Japan, the early implementation of comprehensive oral health coverage led to a decrease in dental caries among children, which in turn reduced the government’s long-term healthcare costs.^9^ Thus, integrating children's oral health into UHC is thus both an ethical imperative and a strategic investment for lifelong health and societal equity.

#### To integrate oral health into the general healthcare system

Delayed diagnosis and treatment can lead to severe ECC, which often requires treatment under general aesthesia for young children. Such care is expensive. Two US studies showed that the average cost of dental treatment under general aesthesia for children ranged from over $5500 in 2008 to $7303 in 2012.^18^ In Canada, day surgery for ECC affected 12.1 out of 1000 children under 6, making up 31% of all day surgeries in this age group.^19^ In Australia, between 2011 and 2012, total of 7890 hospital procedures required general aesthesia for dental reasons in children under 5, accounting for 8.1% of all such procedures.^20^

Unlike medical care, which offers well-child visits since birth, opportunities for routine dental care in early childhood are limited. If parents do not proactively seek dental visits, children often see a dentist only when symptoms arise, leading to more severe and costly oral health problems. Therefore, the WHO advocates for the integration of oral health into primary healthcare services, relying on collaboration among diverse healthcare professionals. However, this integration is still in its early stages.^21^ Various strategies, such as setting up multidisciplinary clinics, establishing a dental home concept, providing paediatric oral healthcare training to all allied healthcare workers, building an interdisciplinary network for data sharing and communication, and employing referral coordinators to close the referral loop, should be implemented to strengthen and support the integration.^22^

#### To initiate policies to advocate for nutritious dietary choices

In 2015, the WHO emphasized the connection between sugar intake levels and the risk of dental caries. The guideline strongly recommends reducing the consumption of free sugars throughout one's lifetime.^23^ Increasing free sugar intake increases caries risk and that limiting free sugar intake to <10% energy intake (E) and to <5% E lowers caries risk.^24^ Therefore, the promotion of healthy diets is a key component of ECC prevention.

One effective strategy is the implementation of a sugar tax. The rationale behind sugar taxation is twofold. Firstly, it serves as a deterrent for consumers, especially price-sensitive individuals, to purchase and consume sugar-sweetened beverages. Introduction of sugar taxes in various countries has led to decreased sales of sugar-sweetened beverages, resulting in positive health outcomes for the population.^25^

Secondly, revenue from sugar taxation can fund public health initiatives for better oral health and healthy diets. This includes oral health promotion campaigns, dental services for underserved populations, and research for ECC prevention. Additionally, it can support broader public health initiatives, such as improving access to healthy food options. Other strategies include regulating advertisements targeting children, healthy school food environments, and mass media for oral health education.

#### To improve physical access to oral health services

Enhancing physical access to oral health services for young children necessitates a comprehensive, multifaceted strategy. One key approach involves increasing the availability of dental clinics in underserved regions to ensure equitable access to oral healthcare. In Japan, for example, the density of dental clinics is notably high, with approximately 67,000 facilities registered as of 2022, according to the Ministry of Health, Labour and Welfare. This equates to roughly 1 dental clinic per 1900 residents.^26^ However, the distribution of these clinics is uneven, with urban areas typically exhibiting higher concentrations than rural localities.

Policy interventions can play a critical role in addressing these disparities. For instance, policymakers may incentivize dental professionals to establish practices in underserved areas through targeted financial incentives such as tax relief or grants. Additionally, investing in infrastructure, particularly in remote or rural areas, lays the groundwork for establishing and sustaining dental services.

Supplementary strategies include the deployment of mobile dental clinics and the implementation of school-based dental programs, which can deliver preventive and therapeutic services directly to children within their communities.^27^ These models reduce logistical barriers and enhance convenience for families. Furthermore, teledentistry presents an innovative solution by facilitating remote consultations, assessments, and referrals, thereby extending the reach of oral health services to children in geographically isolated or resource-limited settings.^28^

Finally, clear referral pathways and protocols should also be established to ensure the timely and appropriate management of ECC cases. In Hong Kong, children under 6 with intellectual disabilities can access the Special Oral Care Service at Hong Kong Children's Hospital through dental units in local public hospitals for timely dental treatment and oral health education.^29^ However, a more comprehensive referral system should be established to further ease access to dental care for all children, ensuring that they receive the necessary care and support for maintaining good oral health.

### Recommendations for international and national dental associations

#### To provide education and interprofessional training

To implement effective continuing education and interprofessional training in paediatric dentistry for ECC management, dental associations should collaborate with dental professionals, primary care providers, healthcare practitioners, and relevant local organizations in program development. Employing a variety of educational methods – such as lectures, workshops, case studies, and hands-on training – can promote interprofessional collaboration and enhance communication among participants. Offering Continuing Professional Development (CPD) points, certificates, or continuing education credits upon program completion can further incentivize participation and support ongoing professional growth.

#### To provide guidelines on ECC management

The American Academy of Pediatric Dentistry and the WHO provide comprehensive recommendations for ECC management, encompassing risk assessment, prevention, early diagnosis, treatment, and follow-up care.^13^^,^^30^ However, the Asia-Pacific region presents unique considerations – such as diverse dietary habits, ethnic backgrounds, and policy environments – that may require adaptation of these guidelines. While regional dental associations contribute to promoting children's oral health, specific, accessible ECC guidelines tailored to local needs are often lacking. To address these distinct challenges, we recommend that regional dental associations develop and disseminate context-specific ECC management guidelines, thereby optimizing oral health outcomes for children across the Asia-Pacific region.

#### To advocate early detection of ECC

ECC is a preventable disease, and early detection is the crucial first step.^31^ International and national dental associations should take responsibility for combating this disease and safeguarding children's oral health. Dental associations can partner with public health agencies and stakeholders to increase ECC awareness and highlight early detection's importance for parents, caregivers, and the public. They can also collaborate with policy makers to promote policies supporting early ECC detection and management. These policies may include better dental care access, insurance coverage for preventive services, and incorporating oral health into primary care.

#### To conduct school-based oral health promotion

Kindergartens offer a familiar and accessible environment for young children, making them an ideal setting for primary healthcare delivery, including dental outreach services. Such programs can provide dental check-ups, preventive care, non-invasive treatments, oral health education, and training for educators. Collaboration with teachers enhances health awareness and promotes proper oral hygiene practices among children. In Hong Kong, a ten-year oral health outreach program reduced ECC prevalence among preschool students from 43% (2010-2011) to 34% (2018-2019).^27^ Similarly, a community-based outreach initiative in Canada improved dental care access and reduced caries prevalence among indigenous children.^32^ Therefore, adopting a proactive approach to children’s oral health is essential. Where resources permit, providing dental hygiene kits – including toothbrushes, toothpaste and dental floss – to preschool children can further promote the establishment of good oral hygiene habits from an early age.

#### To perform annual oral health survey as global surveillance

Surveillance serves as the foundation for public health action by connecting health policies and programs to relevant data. ECC working group experts recommended improving data collection on children's primary teeth in national oral health surveys. They suggested focusing on 5-year-olds and adhering to the diagnosis criteria outlined in the WHO's Oral Health Survey Basic Methods.^33^ Even though the WHO suggests conducting clinical oral health surveys every 5 to 6 years in the same community or setting,^33^ the ECC working group strongly recommends carrying out these surveys annually in each region or country, if the conditions allow.

Standardized data collection allows for meaningful comparisons between different regions and settings. Integrating oral health data with general health surveys can provide valuable insights into associated risk factors and behaviours. Data collection should include: (1) ECC prevalence and experience; (2) demographic information, such as child’s gender, birthdate, and birthplace; (3) oral health-related behaviours, including toothbrushing habits and sugary snack consumption; and (4) socioeconomic indicators, such as family income and parents’ education levels. These data are vital for professionals and policymakers to monitor changes in oral health, identify trends, and evaluate the effectiveness of policies and interventions.

A robust surveillance system for children’s oral health requires data collection from multiple sources, utilizing stratified or random sampling methods to accurately represent diverse populations. Collaboration among stakeholders is essential to facilitate data sharing, capacity building, and evidence-based policy development, while minimizing duplication of efforts.

### Recommendations for individual oral healthcare professionals

#### To adopt early minimal invasive intervention for ECC management

Young children may not cooperate fully during dental treatment. The use of non-invasive or minimally invasive treatment approaches is preferred because they are less resource-demanding, more efficient and cost-effective, and cause less discomfort than other methods.^34^

Placement of pit-and-fissure sealants in molar teeth can reduce the development and progression of new carious lesions into the dentine.^35^ Different types of sealant material have their own merits, but glass-ionomer sealants, which are less demanding on technique and moisture control, are often suitable for use in young children and in community settings.

Topical fluoride products can prevent ECC. Regular application of 5% sodium fluoride varnish can help remineralization of early enamel lesions and has been shown to prevent the development of approximately 37% of new carious lesions in primary teeth.^36^, ^37^, ^38^, ^39^ To maintain its effectiveness, regular applications are necessary every 3 to 6 months. Annual or semi-annual application of 38% silver diamine fluoride solution is effective in arresting caries in primary teeth and in hardening these lesions.^40^ Application of flowable fluoride-releasing glass-ionomer cement to cover the surface of carious dentine lesions can arrest lesions in primary teeth.^41^

These techniques do not require local anesthetic injections and are less invasive, making them more comfortable and “child-friendly.” When choosing the best way to manage ECC lesions, it is important to consider factors such as the country’s oral healthcare system, access to dental professionals and resources, the local community environment, the child’s dental health, how well the child cooperates, and the preferences of the child and their family.

#### To raise awareness on child’s oral health

As an individual dental professional, several proactive measures can be taken to raise awareness about the importance of oral health for young children in managing ECC. The family serves as the child's main resource for understanding health and potential risk factors.^42^^,^^43^ Providing oral health education to parents and caregivers on ECC risk factors may reduce ECC incidence.^44^, ^45^, ^46^ Tooth brushing recommendations and associated behaviours should also be delivered to parents and caregivers.^47^ Firstly, ensure that parents and caregivers are well-informed during appointments by discussing the significance of proper oral hygiene, regular dental check-ups, and a balanced diet for their children. Offer clear guidance on brushing and flossing techniques and emphasize the potential consequences of untreated ECC.

Furthermore, engaging in community outreach is another effective approach.^48^ Participate in local events, such as health fairs or school presentations, to share expertise on children's oral health, and provide free dental screenings or educational materials. Additionally, organizing workshops or seminars tailored for parents, caregivers, and educators, offering practical tips and guidance on maintaining good oral health for young children.^11^ Lastly, using social media to share children's oral health tips, answer questions, and write articles on ECC management for newspapers, websites, and dental publications.

#### To collaborate with healthcare professionals

Forming partnerships with local paediatricians or other healthcare professionals can also be beneficial in promoting the importance of oral health as an integral aspect of a child's overall well-being.^49^ Encourage paediatricians to refer patients for dental check-ups and stay informed about general health issues that may impact a child's oral health.

### Recommendations for academia

#### To conduct research improving child’s oral health

Investing in research to improve ECC management strategies is essential for enhancing the quality of care provided to young children. This research should encompass a wide range of topics, including the effectiveness of various preventive and treatment interventions, the role of social determinants in ECC, and the development of innovative materials and technologies for caries management. Research should also aim to identify best practices for implementing ECC prevention and management programs in diverse settings, taking into account cultural, socio-economic, and geographic factors. Moreover, research capacity should be built in low- and middle-income countries, where the burden of ECC is often greatest.

#### To translate research findings into practice

Collaboration among researchers, dental professionals, and public health authorities is essential for effectively translating research findings on ECC into practical applications and policy recommendations.^50^ To ensure the successful adoption of evidence-based guidelines in dental practices, regular assessments should be conducted to evaluate their effectiveness. This feedback can then be used to refine and enhance the guidelines, ultimately leading to improved implementation, better prevention and treatment outcomes for ECC.

#### To provide education and training opportunities

Academic institutions are essential in providing education and training opportunities for future dental professionals to manage ECC effectively. By updating curricula with the latest progress in ECC management, students or dental professionals can stay informed on current best practices and research findings.^51^ Interdisciplinary collaboration can further enhance the comprehensive approach to ECC prevention and treatment. By focusing on providing the latest education and training opportunities related to ECC management, academic institutions can prepare dental professionals to address this significant oral health issue and improve outcomes for children.

### Recommendations for parents or guardians

#### To encourage proper oral hygiene practices for their children

Parental involvement in their children's oral healthcare can lead to the establishment of lifelong healthy habits and reduced caries risk. Establishing a daily routine for brushing and flossing is important, parents should supervise their children until they can practice these habits independently.^52^

In addition, parents should educate themselves on ECC management, including the use of fluoride toothpaste and dietary recommendations. By practicing good oral hygiene habits themselves, parents can serve as positive role models for their children.^53^

#### To monitor and promote a healthy diet

Parents can promote a healthy diet by providing balanced meals, including fruits, vegetables, whole grains, lean proteins, and low-fat dairy products. Limiting sugary and starchy foods, and encouraging their consumption only at mealtimes, allows for increased saliva production to help neutralize acids and clear food particles from the mouth.^54^ Reducing snacking frequency and encouraging regular meals with enough time between them for saliva to work effectively is also crucial. Furthermore, parents should encourage children to drink water instead of sugary beverages to rinse away food particles, bacteria, and strengthen tooth enamel, ultimately preventing decay.

#### To ensure regular dental check-ups

A child’s first dental visit introduces families to preventive care, giving the dentist a chance to offer guidance on maintaining oral health and to detect any early signs of dental caries. It is recommended that parents schedule regular dental check-ups for their children, beginning with the emergence of the first tooth or, at the latest, by the time they reach 12 months of age.^55^

### Recommendations for educational institutions

#### To promote oral health education into the curriculum

In the Asia-Pacific region, where the enrolment rate of children aged 3 to 5 years in kindergarten is quite high, educational institutions should consider integrating oral health education into their curriculum to effectively manage ECC. One approach is to incorporate oral health lessons into existing health education programs, emphasizing the significance of proper dental hygiene and its impact on overall health. Additionally, arranging role-play activities for children can help raise their awareness of oral health and the importance of maintaining good oral hygiene.^56^

#### To integrate preventive measures for ECC management into daily routines

Some kindergartens have already taken steps to restrict the frequency of snacking and balance the diets of young children.^57^ Implementing fixed times for drinking enough water and incorporating supervised toothbrushing programs, such as organizing children to brush their teeth after school lunch, or providing healthy snacks can further help prevent ECC.

#### To collaborate with dentists and parents for timely children's oral health

Partnering with dental professionals and parents helps to increase awareness and ensure children receive timely access to dental care.^58^ Working together, dentists, educational institutions, and parents can provide the necessary information, resources, and support for children's oral health, ultimately helping to prevent and manage ECC.

## Conclusions

ECC affects about half of preschool children in Asia. However, most preschool children in Asia-Pacific do not receive dental care for their ECC. Oral health is crucial for young children. All stakeholders are responsible for improving oral health for young children in the Asia-Pacific region. The recommendations provide some insights on how to improve the oral health care policies, health care system and oral health care promotion and practice for young children.

## Conflicts of interest

None disclosed.
